# The functional roles of long noncoding RNA DANCR in Human Cancers

**DOI:** 10.7150/jca.44384

**Published:** 2020-10-12

**Authors:** Lei Pan, Xiudi Xiao, Yuan Zhao, Liang Yin, Min Fu, Xu Zhang, Pengcheng Jiang

**Affiliations:** 1 Department of Breast Surgery, The Affiliated People's Hospital of Jiangsu University, 8 Dianli Road, Zhenjiang, Jiangsu 212002, China.; 2 Jiangsu Key Laboratory of Medical Science and Laboratory Medicine, School of Medicine, Jiangsu University, 301 Xuefu Road, Zhenjiang, Jiangsu 212013, China.; 3 Department of General Surgery, The Affiliated People's Hospital of Jiangsu University, 8 Dianli Road, Zhenjiang, Jiangsu 212002, China.

**Keywords:** long noncoding RNA, DANCR, biomarker, therapeutic target

## Abstract

Long noncoding RNAs (lncRNAs) have been wildly explored in various cellular processes and their aberrant expression could lead to tumorigenesis, development and progression. Differentiation antagonizing non-protein coding RNA (DANCR), a well-known lncRNA that is aberrant expression in various tumors, including hepatocellular carcinoma, gastric cancer, colorectal cancer, breast cancer, lung cancer and glioma and so on, in which it functions as oncogene mainly, contributing to cancer development and progression. High expressed DANCR is correlated with poor prognosis. In the present review, we summarize recent progression concerning the role, potential clinical utilities and underlying molecular mechanisms of DANCR related to occurrence and development of multiple cancers.

## Introduction

Cancer is fundamentally caused by genetic alterations that result in the deregulation of the cellular information flow to alter cellular homeostasis and promote growth 1, 2. Most cancers occur as a result of somatic interaction and germline mutations with a variety of environmental factors. And many of these mutations are in regions of the genome that lack protein-coding capacity, yet contain other type of genes that act as RNA molecules, such as the noncoding RNAs 2.

Long noncoding RNAs (lncRNAs) are defined as transcripts of more than 200 nucleotides without obvious protein coding potential 3. The majority of lncRNAs are transcribed by RNA polymerase II, and are capped and polyadenylated at the 5' and 3' ends, respectively 2. Increasing evidence has demonstrated that lncRNAs have tissue-specific expression and are expressed in a regulated manner in various cancers 4. In addition, lncRNAs influence cell cycle regulation, survival, immune response or pluripotency, and other functions in correlation with distinct gene sets, which determine the transformed phenotype of cancer cells 4. Furthermore, several lncRNAs are transcriptionally regulated by key tumor suppressors or oncogenes 2. For example, lncRNA metastasis-associated lung adenocarcinoma transcript 1 (MALAT1) is overexpressed in various tumors, associated with metastasis and invasion, and promotes progression of hepatocellular carcinoma, osteosarcoma and bladder cancer 5-7. In contrast, NF-κB interacting lncRNA (NKILA) is a tumor suppressor and acts as a NF-κB regulator to inhibit breast cancer metastasis 8-10. Taken together, lncRNAs are functional transcripts that contribute to the hallmarks of cancers, and therefore becoming attractive potential therapeutic targets 11-13.

LncRNA differentiation antagonizing non-protein coding RNA (DANCR) also termed as anti-differentiation non-coding RNA (ANCR), which is originally identified a regulator of the suppression of progenitor differentiation 14. Subsequent studies demonstrated that DANCR is overexpressed in many human malignancies, including lung, liver, colon, gastric and breast cancers. In addition, DANCR has been implicated in regulating cancer cell proliferation, apoptosis, migration and invasion and be associated with poor prognosis. This review summarizes the current studies of functions and regulation mechanisms of DANCR in human cancers.

## Discovery of DANCR

In 2011, Kretz et al. discovered an uncharacterized, predicted lncRNA, NR_024031, termed ANCR, was suppressed upon terminal differentiation of multiple cell types 14. The ANCR is an 855-nucleotide lncRNA and its gene locates at chromosome 4, with the closest adjacent annotated genes located 54.8kb upstream of USP46 and 28.7kb downstream from ERVMER34-1 oncogene. The ANCR locus consists of three exons and harbors MIR4449 and SNORA26 in introns 1 and 2, respectively 14. Recent studies have demonstrated that DANCR was overexpressed in major types of cancers, and its expression was closely associated with clinicopathological characteristics, including lymphatic metastasis, distant metastasis and tumor-node-metastasis (TNM) stage. Current evidences suggested that DANCR was associated with important cellular mechanisms and functioned as tumorigenesis, colonization, tumor invasion, metastasis, proliferation, migration, apoptosis, disease progression and prognosis in numerous cancers. The relevant clinicopathological features and underlying molecular mechanisms of DANCR in various cancers are summarized in **Tables 1 and 3.**

## DANCR in various cancers

Accumulating evidences have demonstrated the abnormal expression of DANCR in multiple cancers, including hepatocellular carcinoma, gastric cancer, colorectal cancer, pancreatic cancer, breast cancer, lung cancer, glioma, osteosarcoma, cervical cancer, bladder cancer, prostate cancer, nasopharyngeal carcinoma, esophageal squamous cell carcinoma, ovarian cancer and retinoblastoma. The associations between DANCR expression and the relevant clinicopathological features of various cancers are summarized in **Table 1.** And the potential as biomarkers for diagnosis and prognosis of DANCR in cancers are presented in **Table 2.** The functions and underlying molecular mechanisms of DANCR in various cancers are summarized in **Table 3** and detailed in the rest of this review.

### Hepatocellular carcinoma

Yuan et al. found that DANCR was overexpressed in stem-like hepatocellular carcinoma cells and associated with CTNNB1, which could block the repressing effect of miR-214, miR-320a and miR-199a 15. And DANCR was up-regulated in tumor tissues and plasma of patients with hepatocellular carcinoma, and plasma DANCR might be a useful biomarker for hepatocellular carcinoma diagnosis. DANCR expression was highly correlated with microvascular and liver capsule invasion of hepatocellular carcinoma. Functionally, knockdown of DANCR inhibited hepatocellular carcinoma cell proliferation and invasion by suppressing β-catenin signaling 16. Silencing DANCR expression could promote hepatocellular carcinoma cell apoptosis, meanwhile cell cycle progression was blocked in G1 phase. DANCR could contribute to hepatocellular carcinoma malignancy via sponging miR-216a-5p and modulating KLF12 17. In addition, Guo et al. reported that DANCR could decoy miR-27a-3p to regulate ROCK1/LIMK1/COFILIN1 pathway to promote hepatocellular carcinoma progression 18.

### Gastric cancer

Hao et al. confirmed that DANCR was dramatically upregulated in gastric cancer tissues and DANCR overexpression was significantly associated with worse overall survival in gastric cancer patients. Their experiments revealed that overexpression of DANCR notably increased gastric cancer cell proliferation by influencing the gene expression programs in cell metabolic and cycle process 19. DANCR overexpression was significantly correlated with lymph node metastasis and late clinical stage. LncRNA-LET was a target gene of DANCR, and DANCR could associate with enhancer of zeste homolog 2 (EZH2) and HDAC3 to epigenetically silence lncRNA-LET, then regulate gastric cancer migration and invasion 20. Otherwise, our previous research showed that the expression of DANCR was higher in the serum of gastric cancer patients. DANCR was upregulated by SALL4 and exerted its tumor-promoting roles partly through the activation of β-catenin pathway 21. Further research showed that DANCR expression remained high in cisplatin (DDP) resistant gastric cancer tissues or cells. DANCR overexpression showed increased survival and decreased apoptosis. In addition, DANCR overexpression could upregulate the expression of MDR1 and MRP1 to accelerate the multidrug resistance (MDR) of gastric cancer 22.

### Colorectal cancer

In 2015, Liu et al. reported DANCR expression was increased in colorectal cancer tissues compared with that in adjacent normal tissues. And high DANCR expression was correlated with TNM stage, histologic grade, and lymph node metastasis. They found that DANCR expression was an independent poor prognostic factor for both overall survival (OS) and disease-free survival (DFS) 23. Zhao et al. found that ANCR and EZH2 were highly expressed in colorectal cancer tissues, and correlated with lymphatic metastasis, Dukes stage and TNM stage. Their results suggested that ANCR could influence the invasion and migration of colorectal cancer cells by specifically binding to EZH2 24. Wang et al. reported that DANCR and heat shock protein 27 (HSP27) were both targets of miR-577 and shared the same binding site. DANCR could promote HSP27 expression and its mediation of proliferation/metastasis via miR-577 sponging 25.

### Pancreatic cancer

Chen et al. revealed that DANCR could promote pancreatic ductal adenocarcinoma progression, with relatively higher expression levels in pancreatic ductal adenocarcinoma cell lines and tissues. Correlation analysis of clinicopathological features and DANCR expression found that high DANCR expression was statistically correlated with vascular invasion, advanced T stage, lymph node metastasis and advanced TNM stage. High DANCR expression was identified as an independent risk factor for poor OS and progression-free survival (PFS) of pancreatic ductal adenocarcinoma. DANCR could promote the proliferation and metastasis of pancreatic ductal adenocarcinoma cells. Besides, miR-33a-5p/AXL signaling pathway may be involved in mediating the function of DANCR 26. Yao et al. reported that DANCR was markedly upregulated in clinical tissues and cell lines of pancreatic cancer. High DANCR expression exhibited a significant correlation with poor prognosis. And DANCR could promote pancreatic cancer cell growth and metastasis through working as a competing endogenous RNA (ceRNA) of miR-214-5p, and positively regulate E2F2 expression in pancreatic cancer cells 27. Otherwise, A negative relationship was found between DANCR and miR-135a expression in pancreatic cancer. DANCR was downregulated by miR-135a through regulating of downstream protein NLRP3 in pancreatic cancer 28.

### Breast cancer

Li et al. initially identified that ANCR could potentiate the CDK1-EZH2 interaction, which then increase the intensity of phosphorylation at Thr-345 and Thr-487 sites of EZH2, facilitating EZH2 ubiquitination and hence its degradation. They uncovered that ANCR level was lower in breast cancer tissues and breast cancer cell lines, in contrast to their normal counterparts. ANCR is an important player in breast cancer progression and metastasis mainly through decreasing EZH2 stability 29. TGF-β participates in epithelial-mesenchymal transition (EMT) and involves in physiological and pathological functions of tumor progression. Li et al. confirmed that ANCR participated in TGF-β1-induced EMT and TGF-β1 could down-regulate ANCR expression by increasing HDAC3 enrichment at ANCR promoter region, which decrease both H3 and H4 acetylation of ANCR promoter in breast cancer. Downstream, ANCR could inhibit breast cancer cell migration and breast cancer metastasis by decreasing RUNX2 *in vitro* and *in vivo*. In addition, ectopic expression of ANCR partly attenuated the TGF-β1-induced EMT. Therefore, as a new TGF-β downstream molecular, ANCR is essential for TGF-β1-induced EMT by decreasing RUNX2 expression 30.

While, Sha, Tao and Tang et al. identified that DANCR was significantly overregulated in triple negative breast cancer tissues and cell lines 31-33. Kaplan-Meier analysis showed that higher DANCR expression was correlated with worse TNM stages and a shorter OS. Further study indicated that DANCR overexpression significantly promoted cell proliferation and invasion *in vitro* and contributed to tumor growth *in vivo*. Sha et al. revealed that DANCR knockdown was associated with increased binding of EZH2 on the promoters of CD44 and ABCG2, and concomitant reduction of expression of these genes, suggesting that they may be DANCR targets in triple negative breast cancer 31. Tao et al demonstrated that miRNA-216a-5p was a target of DANCR by bioinformatics analysis. Their experiments demonstrated that miRNA-216a-5p interacted with DANCR and its inhibitor could weaken the influences induced by DANCR knockdown for cancer cells, including cell proliferation and invasion, and the expression of Nanog, SOX2 and Oct4 32. In addition, Tang et al. demonstrated that DANCR bound with RXRA and increased its serine 49/78 phosphorylation via GSK3β, resulting in activating PIK3CA transcription, and subsequently enhanced PI3K/AKT signaling and triple negative breast cancer tumorigenesis 33.

### Lung cancer

Zhen et al. saw DANCR upregulation in lung cancer, particularly in high-grade lung cancer tissues and aggressive cancer cells. The DANCR overexpression promoted cell proliferation and colony formation *in vitro*, and DANCR interference effectively suppressed lung cancer progression both *in vitro* and *in vivo*. They found that the miR-216a level in cancer cells was negatively correlated with DANCR expression 34. Lu et al. demonstrated that DANCR was up-regulated in lung adenocarcinoma and that DANCR knockdown inhibited tumor cell proliferation, migration and invasion and restored cell apoptosis rescued. They found that mTOR was a target of miR-496 and that DANCR could modulate the expression levels of mTOR by working as a ceRNA 35. In addition, elevated DANCR expression was associated with poor prognosis in the patients with lung adenocarcinoma. And DANCR promoted the invasive ability via regulation of high mobility group AT-hook 2 (HMGA2) in lung adenocarcinoma cells, SPCA1 and A549 36.

The study of Wang revealed that DANCR was markedly upregulated in non-small-cell lung cancer tumor tissues and cell lines compared with related normal controls. DANCR could function as a tumor promoter and promote cell proliferation, invasion and migration through directly targeting the tumor suppressor miR-758-3p in non-small-cell lung cancer 37. The upregulation of DANCR expression was significantly associated with larger tumor size, advanced TNM stage and lymph node metastasis, and also predicted poor prognosis of patients with non-small-cell lung cancer. DANCR could compete with Sox4 mRNA to bind with miR-138, thus affecting Sox4 expression. In addition, Sox4 could bind to the promoter regions of DANCR gene to activate DANCR expression, suggesting a positive feedback loop of DANCR/miR-138/Sox4 in non-small-cell lung cancer 38. Guo et al. revealed that the knockdown of DANCR inhibited non-small-cell lung cancer cell proliferation by inducing cell apoptosis and cell cycle arrest. In addition, DANCR knockdown suppressed non-small-cell lung cancer cell migration and invasion via inhibition of EMT. DANCR knockdown inhibited EZH2-mediated epigenetic silencing of p21 promoter and increased p21 expression 39. While, Wang et al. found that ANCR was significantly downregulated in non-small-cell lung cancer patients compared with healthy controls in lung biopsies and plasma. Downregulated expression of ANCR distinguished non-small-cell lung cancer patients from healthy controls and low non-small-cell lung cancer expression level indicated shorter postoperative survival time of non-small-cell lung cancer patients. ANCR inhibited non-small-cell lung cancer cell migration and invasion, at least partially by downregulating TGF-β1 expression 40.

### Glioma

Yang et al. explored that DANCR was increased in glioma tissues and cells compared with normal brain tissues and cells. DANCR expression was positively correlated with the malignancy and poor prognosis of glioma patients. DANCR contained a binding site of miR-33a-5p and DANCR expression was negatively correlated with the expression of miR-33a-5p in glioma tissues. DANCR could facilitate cancer cell proliferation, invasion and metastasis and inhibit apoptosis through competitively binding to miR-33a-5p in glioma 41. High DANCR expression was correlated with advanced tumor grade. Inhibition of DANCR suppressed the glioma cell proliferation and induced cell arrested in the G0/G1 phase. DANCR could directly interact with miR-634 in glioma cells and this interaction results in the inhibition of downstream of RAB1A expression 42. Li et al. verified that DANCR positively affected glioma progression via activating Wnt/β-catenin signaling pathway 43. In addition, DANCR expression was negatively correlated with cisplatin sensitivity in glioma cells. DANCR could attenuate cisplatin-induced cell proliferation inhibition *in vitro*, xenograft growth suppression *in vivo* and cisplatin-induced cell apoptosis *in vitro* and *in vivo*. DANCR upregulated AXL via competitively binding miR-33a-5p, miR-33b-5p, miR-1-3p, miR-206 and miR-613. Through upregulating AXL, DANCR activated PI3K/Akt/NF-κB signaling pathway in glioma cells. DANCR could promote cisplatin resistance via activating AXL/PI3K/Akt/ NF-κB signaling pathway in glioma 44.

### Osteosarcoma

The present study of Jiang et al. showed that DANCR was significantly increased in osteosarcoma tissues, and its expression was positively correlated with tumor size and metastasis as an independent poor prognostic factor. The overexpression of DANCR could increase osteosarcoma cell proliferation, migration, and invasion *in vitro*, as well as promote xenograft tumor growth and lung metastasis *in vivo*. DANCR upregulated expression of the receptor tyrosine kinase AXL by competitively binding to miR-33a-5p, which targets AXL mRNA for degradation, and this function was sequentially performed through the PI3K-AKT signaling pathway 45. Wang et al. firstly demonstrated that DANCR could decoy two miRNAs — miR-335-5p and miR-1972 to facilitate ROCK1-mediated proliferation and metastasis in osteosarcomab 46.

Li et al. found that ANCR was highly expressed in human osteosarcoma cell lines. And their data indicated that ANCR might be an oncogenic lncRNA that promoted proliferation of osteosarcoma 47. ANCR depletion inhibited the proliferation, invasion, and migration of osteosarcoma cells. Downregulation of ANCR could decrease the abundance of EZH2 and activate the expression of both p21 and p27. The interaction between ANCR with EZH2 indicated that ANCR might exert its function via binding to EZH2 48.

### Cervical cancer

In Liang's study, they discovered that DANCR was significantly elevated in cervical cancer tissues and cells, and was closely correlated with poor prognosis of cervical cancer patients. In addition, knockdown of DANCR inhibited cervical cancer cell proliferation, migration, and invasion *in vitro*, indicating that DANCR functioned as an oncogene in cervical cancer. They verified that DANCR could regulate ROCK1 expression by competitively binding to miR-335-5p 49. In addition, Cao et al. identified miR-665 as the ceRNA for DANCR, and their observations suggested that DANCR-mediated miR-665 downregulation could regulate the malignant phenotype of cervical cancer cells by targeting transforming growth factor beta receptor 1 (TGFBR1) through the ERK/SMAD pathway 50.

### Bladder Cancer

Zhao et al. found that DANCR was significantly up-regulated in bladder cancer, and increased DANCR expression was positively correlated with higher histological grade and advanced TNM stage. Knockdown of DANCR could inhibit malignant phenotypes and EMT of bladder cancer cells. And they found that DANCR was distributed mostly in the cytoplasm and functioned as a miRNA sponge to positively regulate the expression of musashi RNA binding protein 2 (MSI2) through sponging miR-149 and subsequently promoted malignant phenotypes of bladder cancer cells 51. Chen et al discovered that DANCR was significantly upregulated in cases with lymph node metastasis. DANCR expression was positively correlated with lymph node metastasis status, tumor stage, histological grade, and poor patient prognosis. Functional assays demonstrated that DANCR promoted bladder cancer cell migration, invasion, and proliferation *in vitro* and enhanced tumor lymph node metastasis and growth *in vivo*. DANCR could activate IL-11-STAT3 signaling and increase cyclin D1 and PLAU expression via guiding leucine-rich pentatricopeptide repeat containing (LRPPRC) to stabilize mRNA 52.

### Prostate cancer

In 2015, Jia et al. found the expression of DANCR was increased in prostate cancer tissues and cells compared to normal prostate cancer tissues and cells. Moreover, DANCR promoted prostate cancer cell invasion and migration *in vitro* and metastasis of tumor xenografts in nude mice. They found that TIMP2/3, which are critical metastasis inhibitor of prostate cancer, were down-regulated by DANCR synergistically with EZH2 through epigenetically silencing their promoter. DANCR expression was repressed by androgen-AR signaling pathway and DANCR knockdown facilitated the upregulation of TIMP2/3 and the suppression of invasion and migration by androgen-AR, while DANCR knockdown decreased the promotion of invasion and migration in prostate cancer cells by enzalutamide treatment 53. Ma et al. reported that DANCR was significantly upregulated in docetaxel (DTX) resistant prostate cancer. Silencing of DANCR could improve the DTX efficacy in DTX-resistant prostate cancer cells. DANCR served as a ceRNA of miR-34a-5p, leading to the derepression of miR-34a-5p target JAG1 to enhance DTX resistance of prostate cancer 54.

### Nasopharyngeal carcinoma

Wen et al. reported that DANCR was upregulated in nasopharyngeal carcinoma, especially in those with lymph lode metastasis, and its upregulation could predict poor survival. And DANCR was responsible for nasopharyngeal carcinoma metastasis and hypoxia phenotype. DANCR could stabilize HIF-1α mRNA through interacting with NF90/NF45 complex, leading to nasopharyngeal carcinoma metastasis and invasion 55. Hao's study suggested that DANCR overexpression promoted nasopharyngeal carcinoma cell proliferation and migration and inhibited apoptosis. DANCR could regulate the phosphorylation of AKT serine/threonine kinase and the protein expression of PTEN in nasopharyngeal carcinoma cells 56. In addition, Zhang et al. identified that DANCR was highly expressed in nasopharyngeal carcinoma cells and IL-6 stimulation upregulated DANCR expression and invasion of nasopharyngeal carcinoma cells. Their study demonstrated that DANCR, acting as an oncogene in nasopharyngeal carcinoma, promoted nasopharyngeal carcinoma progression by interacting with STAT3 and enhancing JAK1 binding to STAT3 to strengthen IL-6/JAK1/STAT3 signaling^57^. Ma et al. identified that ANCR was upregulated in nasopharyngeal carcinoma tissues and cells. ANCR expression was significantly correlated with tumor size and TNM stage. ANCR could promote nasopharyngeal carcinoma cell growth and radiation resistance by repressing the expression of PTEN. And this regulation relied on ANCR-mediated EZH2 binding and epigenetic regulation on PTEN promoter 58.

### Other cancers

In recent days, some studies also reported that DANCR played important role in other cancers such as papillary thyroid cancer, esophageal squamous cell carcinoma, ovarian cancer and retinoblastoma.

Zhang et al. demonstrated that the expression of DANCR was notably decreased in papillary thyroid cancer tissues in comparison with adjacent normal tissues. DANCR expression level was correlated to T grade and TNM stage. Their results suggested that DANCR was an independent factor for TNM stage of papillary thyroid cancer 59. Shi et al. demonstrated that the expression of DANCR in esophageal squamous cell carcinoma tissues was significantly higher compared with that in the adjacent normal tissues. Furthermore, cell proliferation, migration and invasion were significantly suppressed, while cell apoptosis was promoted by knockdown mediated downregulation of DANCR expression 60. Gao et al. revealed that the expression of DANCR in ovarian cancer samples was significantly higher than that of the corresponding normal tissues. DANCR could enhance the proliferation, migration and invasion capacities of ovarian cancer cells by upregulating insulin-like growth factor 2 (IGF2) 61. Wang et al. revealed that DANCR was upregulated in retinoblastoma tissues and cell lines. The ectopic overexpression of DANCR indicated poor overall survivals and DFS for retinoblastoma patients. DANCR could function as ceRNA for miR-34c and miR-613 to modulate progression and metastasis in retinoblastoma oncogenesis via targeting MMP-9 62.

## Regulatory mechanisms of DANCR in human cancers

**Figure 1:**
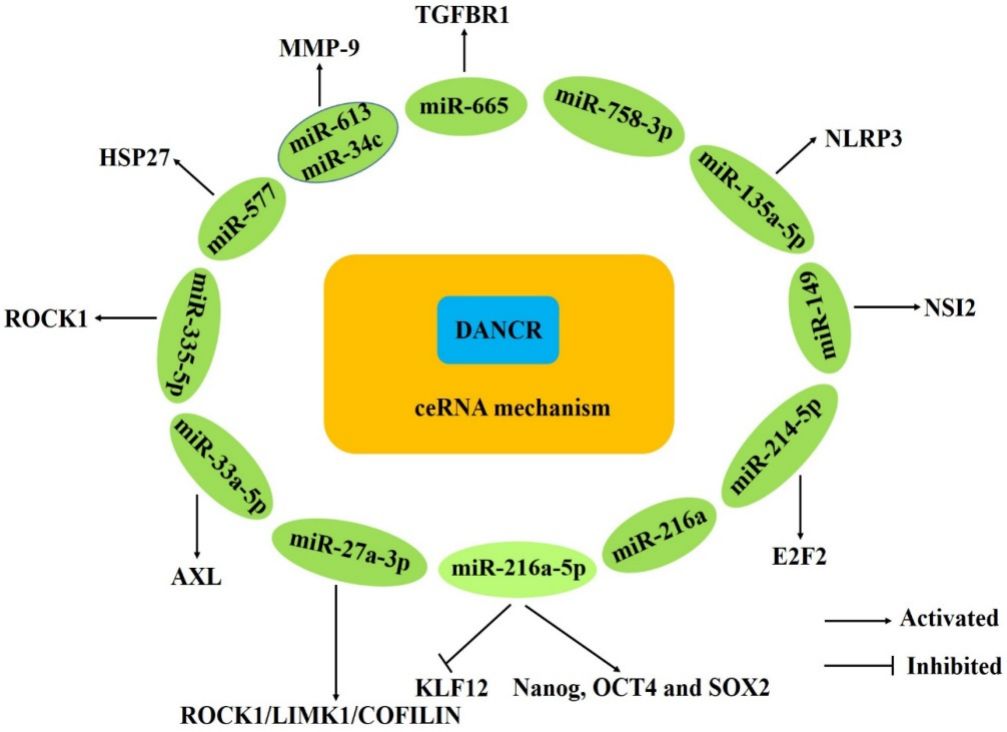
DANCR acts as ceRNAs in human cancers.

**Figure 2:**
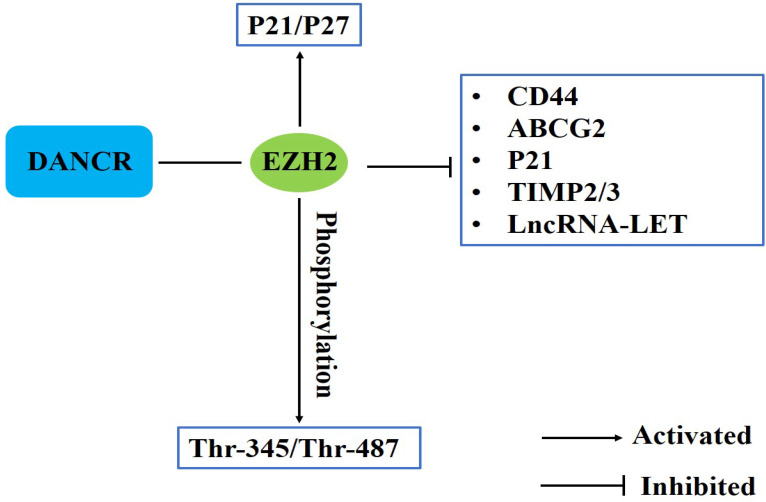
DANCR involves in epigenetic regulation in human cancers.

**Figure 3:**
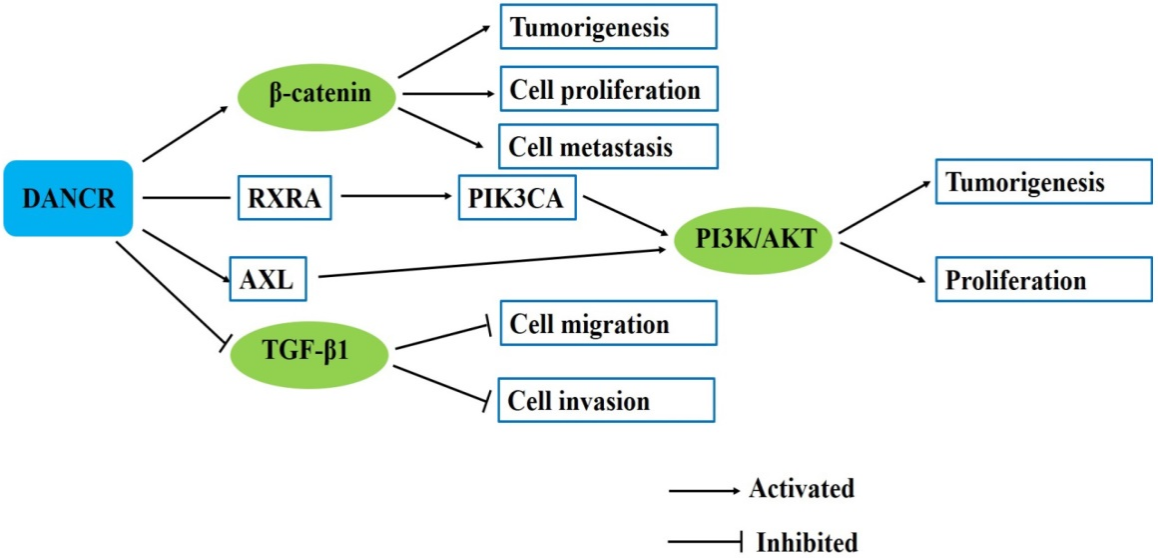
DANCR involves in several signaling pathways in human cancers.

**Figure 4:**
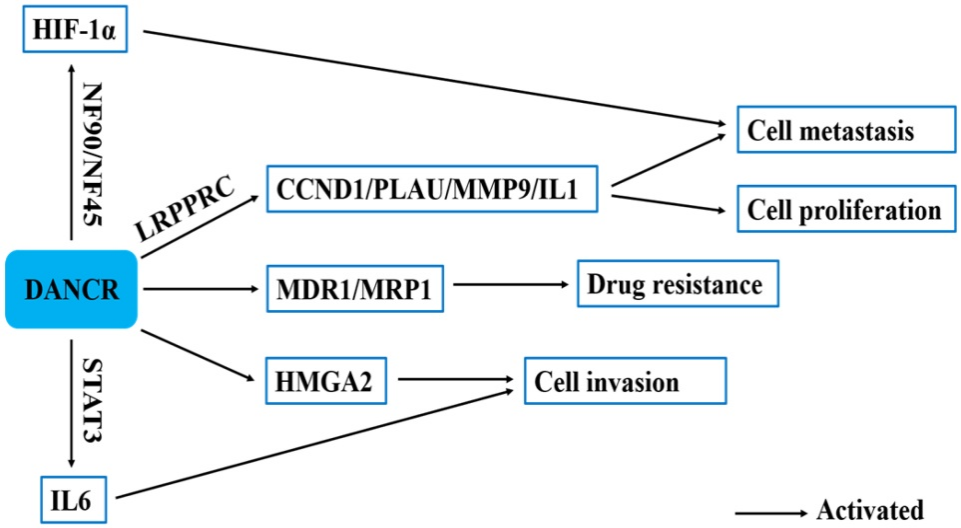
The other regulatory mechanisms of in human cancers.

DANCR abnormal expression in various human cancers has been shown to play active roles through regulating cancer-related phenotypes, as promoting cell proliferation, migration, invasion, inhibiting apoptosis. Furthermore, the regulatory mechanisms of DANCR are complex and involve multiple factors (**Figs. 1-4**).

### Acting as a ceRNA

CeRNAs is a novel regulatory mechanism whereby lncRNAs function as ceRNAs to sponge microRNAs and simultaneously target both noncoding RNAs and genes 63, 64. Several articles have confirmed that DANCR could function as a ceRNA to regulate the expression of specific genes, exerting its oncogenic function in various cancers (**Fig. 1**). Wang and Tao et al. demonstrated that DANCR could directly interact with miR-216a-5p to regulate the expression of KLF12, Nanog, OCT4 and SOX2 17, 32. Guo et al. found that DANCR could promote hepatocellular carcinoma development by decoying miR-27a-3p to regulate ROCK1/LIMK1/COFILIN pathway 18. Wang et al. revealed that DANCR could promote HSP27 expression and its mediation of proliferation/metastasis via miR-577 sponging in colorectal cancer 25. The DANCR overexpression promoted cell proliferation, colony formation, migration and invasion by interacting with miR-216a and miR-758-3p and modulated mTOR expression through directly binding to miR-496 in lung cancer 34, 35, 37. Yao et al. revealed that DANCR could positively regulate E2F2 expression through working as a ceRNA of miR-214-5p in prostate cancer cells 27. And DANCR regulated the expression of E-cadherin, N-cadherin and NLRP3 by targeting miR-135a-5p in prostate cancer 28. DANCR could facilitate glioma malignancy and upregulate AXL to increase osteosarcoma cells function via competitively binding to miR-33a-5p 41, 45. Xu et al. showed that DANCR directly interacted with miR-634 and this interaction inhibited the downstream of RAB1A expression in glioma 42. Wang et al. and Liang et al. verified that DANCR could regulate ROCK1 expression by competitively binding to miR-335-5p 46, 49. Cao et al. proved that DANCR could directly interact with miR-665 to regulate proliferation and metastasis of cervical cancer through the ERK/SMAD pathway 50. Zhan et al. found that DANCR positively regulated the expression of MSI2 though sponging miR-149 and subsequently promoted malignant phenotypes of breast cancer cells 51. In addition, Wang et al. revealed that DANCR could upregulate the MMP-9 protein expression by competitively binding to miR-34c and miR-613 62.

### Epigenetic regulation

EZH2 is one component of Polycomb Repressive Complex (PRC2), which catalyzes the trimethylation of Lys-27 of histone H3 (H3K27me3) to suppress the transcription of target genes. EZH2, as a key epigenetic regulator, participates in a variety of cancer metastasis 29. Current researches have demonstrated that DANCR could interact with EZH2 to epigenetically silence CD44, ABCG2, p21 and TIMP2/3 expression 31, 39, 53. Mao et al. revealed that DANCR could function as a molecular scaffold of EZH2 and HDAC3 to epigenetically silence another lncRNA, lncRNA-LET 20. ANCR could specifically bind to EZH2 and activate p21, p27 expression in osteosarcoma and influence the invasion and migration of colorectal cancer cells 24, 48. Li et al indicated that ANCR-EZH2 binding might facilitate CDK1 interaction with EZH2 to promote phosphorylation at Thr-345 and Thr-487 residues of EZH2, and finally potentiate EZH2 ubiquitination degradation through ubiquitin-proteasome pathway 29 (**Fig. 2**).

## Participation in signaling pathways

### β-catenin signaling pathway

Previous reports indicated that the activation of β-catenin pathway promoted tumor cell proliferation and metastasis 16. For hepatocellular carcinoma, recently, Yuan et al. indicated that DANCR increased stemness feature of hepatocellular carcinoma via derepression of catenin beta 1 (CTNNB1) 15. Ma et al. found that the activity of β-catenin was significantly reduced by siDANCR and genes downstream of β-catenin, namely vimentin, CCND1 and MYC were all downregulated by siDANCR in hepatocellular carcinoma cells 16. Our previous study found that DANCR knockdown decreased while DANCR overexpression increased the expression of β-catenin in gastric cancer cells. And the increased expression of β-catenin and its target gene c-Myc by DANCR was inhibited by β-catenin siRNA in gastric cancer cells. DANCR could activate β-catenin to promote gastric cancer progression 21. In addition, western blot assay was conducted in glioma cells to demonstrate that the protein levels of the Wnt/β-catenin pathway related proteins were negatively modulated by si-DANCR 43. These findings suggest that DANCR could activate the β-catenin signaling pathway to act oncogenic roles in various cancers (**Fig. 3**).

### PI3K signaling pathway

The PI3K pathway plays a key role in the regulation of multiple cellular events, including cell growth, proliferation, cell cycle progression and survival 65. Tang et al. showed that DANCR directly bound with RXRA and mediated its phosphorylation. RXRA could bind with the promoter of PIK3CA and downregulate PIK3CA transcription. DANCR knockdown inhibited RXRA protein phosphorylation and PIK3CA expression level. DANCR-induced RXRA phosphorylation suppressed RXRA-inhibited PIK3CA transcription in triple negative breast cancer cells and ultimately activated the downstream PI3K/AKT signaling 33. AXL is an important receptor tyrosine kinase, which activates PI3K/AKT/NF-κB signaling pathway. Ma et al. found that through upregulating AXL, DANCR increased phosphorylation levels of AKT and IκBα, and activated PI3K/AKT/NF-κB signaling pathway 44. These findings suggest that DANCR could promote cancer progression and mediate drug resistance by activating PI3K/AKT signaling pathway (**Fig. 3**).

### TGF-β signaling pathway

TGF-β signaling pathway interacts with multiple signaling pathways to participate in the development and progression of different types of human malignancies 66. TGF-β acts as a tumor suppressor in the early stage of tumor development by inhibiting cell proliferation and inducing EMT to promote tumor cell invasion 67, 68. Li et al. revealed that TGF-β1 down-regulated ANCR expression by increasing HDAC3 enrichment at ANCR promoter region, which decreased both H3 and H4 acetylation of ANCR promoter. Western blot and transwell assays indicated that ectopic expression of ANCR partly attenuated the TGF-β1-induced EMT 30. Wang et al. indicated that ANCR overexpression could inhibit non-small-cell lung cancer cell migration and invasion and downregulated TGF-β1 expression, while TGF-β1 treatment showed no significant effects on ANCR expression but promoted non-small-cell lung cancer cell migration and invasion. TGF-β1 treatment could attenuate the inhibitory effects of ANCR overexpression on non-small-cell lung cancer cell migration and invasion 40 (**Fig. 3**).

### Other mechanisms

Xu et al. found that DANCR overexpression markedly upregulated mRNA and protein levels of MDR-related genes, such as MDR1 and MRP1 to contribute to the development of MDR 22. Zhang et al. revealed that DANCR knockdown inhibited HMGA2 expression and the inhibition of HMGA2 suppressed the invasive ability of lung adenocarcinoma cells. DANCR could promote the invasion of lung adenocarcinoma cells by positively regulating HMGA2 36. LRPPRC is an RNA-binding protein that regulates mRNA stability and polyadenylation 52. DANCR could directly interact with LRPPRC, LI-11, PLAU and CCND1 mRNA. DANCR guided LRPPRC to stabilize IL-11, PLAU, and CCND1 mRNAs, which activated IL-11-STAT3 signaling and enhances CCND1 and PLAU expression levels 52. Zhang et al. revealed that IL-6 promoted DANCR transcription partly by activating STAT3 phosphorylation. Besides, DANCR strengthened IL-6 stimulation-induced STAT3 phosphorylation by interacting with STAT3. There is an obviously existing positive feedback between STAT3 phosphorylation and DANCR transcription. DANCR could promote STAT3 activation by facilitating JAK1 to phosphorylate STAT3. DANCR could interact with STAT3 and enhance JAK1 binding to STAT3 to strengthen IL-6/JAK1/STAT3 signaling 57. NF90, as a double-stranded RNA-binding protein, is transcribed from the interleukin enhancer binding factor 3 (ILF3) gene. Studies demonstrated that the NF90/NF45 complex can regulate gene expression and mRNA stability and play an important role in RNA metabolism and viral replication. DANCR could stabilize HIF-1α mRNA through interacting with NF90/NF45 complex, leading to nasopharyngeal carcinoma metastasis 55 (**Fig. 4**).

## Conclusions and Future perspectives

With the repaid development of next-generation sequencing technology, lncRNAs have been identified dysregulated as oncogenes or tumor suppressor genes, which play important regulatory roles in tumor formation and development. Since its discovery, DANCR has been widely investigated in various human malignancies. DANCR is overexpressed and plays an oncogenic role in most types of tumors, including breast cancer and non-small-cell lung cancer. But Li et al. found that ANCR was downregulated in breast cancer and in particular, they demonstrated that a variable ANCR expression pattern and biological function in different cancer types and different cell lines 29. Besides, Wang et al. suggested that ANCR was downregulated in non-small-cell lung cancer 40. Whether the expression of DANCR is increased or decreased in breast cancer and lung cancer remains to be further detected. DANCR expression in different subtypes of breast cancer and lung cancer might be different. Amplified DANCR is dramatically correlated with multiple clinicopathological features and prognosis, such as TNM stage, lymph node metastasis and over survival. Furthermore, DANCR is included in cellular functions such as cell proliferation, migration, invasion and cell apoptosis, suggesting that DANCR contributes to tumorigenesis and tumor progression. Li and Wang et al. manifested that ANCR could function as a tumor suppressor 30, 40. ANCR was likely to be involved in the metastasis but not the growth of non-small-cell lung cancer 40. The expression of ANCR might be related to different phenotypes of cancer cells. The functional roles of DANCR in different cancers might be further verified. The underlying molecular mechanisms of DANCR involved in multiple cancers have been explored preliminarily. DANCR could function as ceRNA in the regulation of specific gene expression by competing for specific miRNAs and interact with some proteins to regulate gene expression in epigenetic regulation. And DANCR is demonstrated to take part in several signaling pathways contributing to carcinogenesis and cancer progression, including β-catenin signaling pathway, PI3K signaling pathway and TGF-β signaling pathway.

The molecular functions and cellular mechanisms by which DANCR mediates its actions are complicated and associated with multiple factors. Several experiments have been identified that lncRNAs play a role in epigenetic, transcriptional, and post-transcriptional levels to contribute carcinogenesis and cancer development. Therefore, the molecular mechanism of DANCR in each cancer type should be clarified. Recently, lncRNAs have been demonstrated not only in tumor tissues but also in body fluids including plasma, serum, urine, and so on. The expression of DANCR in body fluids including plasma should be detected. And multiple effects between DANCR and molecular target markers should be explored in most available diagnostic samples like blood to apply it in clinical use. Further studies on the clinical value of DANCR in the diagnosis and treatment of cancers require more attention.

## Figures and Tables

**Table 1:** Clinical significance of DANCR in various tumors

Cancer type	Relevant clinicopathological features	Clinical value	References
Hepatocellular carcinoma	Microvascular and liver capsule invasion, tumor growth, lung metastasis	Diagnostic biomarker, therapeutic target, prognostic marker	15, 16, 18
Gastric cancer	Worse overall survival, lymph node metastasis, advanced TNM stage, tumor size, invasion depth, cisplatin resistant, poor prognosis	Diagnostic biomarker, therapeutic target, prognostic marker	19, 21, 22
Colorectal cancer	Worse overall survival, worse disease-free survival, lymph node metastasis, advanced TNM stage, histologic grade, Duckes stage, poor prognosis	Therapeutic target, prognostic marker	23-25
Pancreatic cancer	Vascular invasion, advanced T stage, advanced TNM stage, lymph node metastasis, lower OS rate, shorter PFS period, poor prognosis	Therapeutic target	27, 28
Breast cancer	Advanced TNM stage, shorter OS	Diagnostic biomarker, therapeutic target, prognostic marker	30-32
Lung cancer	Larger tumor size, advanced TNM stage, lymph node metastasis, shorter postoperative survival time, poor prognosis	Diagnostic biomarker, therapeutic target, prognostic marker	36, 38-40
Glioma	Malignancy, advanced tumor grade, poor prognosis, cisplatin sensitivity	Therapeutic target, biomarker for predicting cisplatin sensitivity	41, 42, 44
Osteosarcoma	Tumor size, tumor growth, lung metastasis, poor prognosis,	Therapeutic target, prognostic marker	45, 46
Cervical cancer	Malignant phenotype, poor prognosis	Therapeutic target	49, 50
Bladder cancer	Higher histological grade, advanced TNM stage, lymph node metastasis, tumor stage, poor prognosis,	Diagnostic biomarker, therapeutic target	51, 52
Prostate cancer	Docetaxel resistant	Target for preventing prostate cancer metastasis	53
Nasopharyngeal carcinoma	Lymph node metastasis, poor survival, hypoxia phenotype, tumor size, TNM stage, radiation resistance	Therapeutic target, prognostic marker, biomarker and therapeutic target for radiation resistance	55, 56, 58
Papillary thyroid cancer	T grade, TNM stage	Diagnostic biomarker,	59
Retinoblastoma	Poor overall survival and disease-free survival	-	62

**Table 2:** DANCR in tissues/serum/plasma as diagnostic and prognostic biomarkers

Cancer type	Biomarker type	Specificity	Sensitivity	AUC	Sample	References
Hepatocellular carcinoma	Diagnostic/prognostic	84.3%	80.8%	0.868	Plasma	15, 16
Gastric cancer	Diagnostic/prognostic	67.7%	64.6%	0.704	Tissues	19, 21
		79.5%	72.7%	0.816	Serum	
Colorectal cancer	Prognostic	NA	NA	NA	Tissues	23
Breast cancer	Diagnostic/prognostic	NA	NA	NA	Tissues	30, 31
Lung cancer	Diagnostic/prognostic	NA	NA	0.927	Tissues	39, 40
		NA	NA	0.883	Plasma	
Osteosarcoma	Prognostic	NA	NA	NA	Tissues	46
Bladder cancer	Diagnostic	NA	NA	NA	Tissues	51
Nasopharyngeal carcinoma	Diagnostic/prognostic	NA	NA	NA	Tissues	55, 58
Papillary thyroid cancer	Diagnostic	66.2%	85.3%	0.823	Tissues	59
		82.2%	81.5%	0.876		
		91.7%	83.3%	0.917		

**Table 3:** Functional characterization of DANCR in various tumors

Cancer types	Expression	Role	Functional role	Related genes/pathway	References
Hepatocellular carcinoma	Overexpression	Oncogene	Proliferation, invasion, metastasis, apoptosis, cell cycle, EMT progression, stemness feature, colonization	β-catenin pathway, miR-216a-5p, KLF2, miR-27a-3p, miR-214, miR-320a, miR-199a, CTNNB1	15-18
Gastric cancer	Overexpression	Oncogene	Proliferation, invasion, metastasis, apoptosis, cell metabolic, cell cycle, EMT progression, multidrug resistance	EZH2, HDAC3, lncRNA-LET, SALL4, β-catenin pathway, MDR1, MRP1	19-22
Colorectal cancer	Overexpression	Oncogene	Proliferation, invasion, metastasis	EZH2, H3K27me3, miR-577, HSP27	23-25
Pancreatic cancer	Overexpression	Oncogene	Proliferation, invasion, metastasis	miR-33a-5p, AXL, miR-214-5p, E2F2, miR-135a, NLRP3	26-28
Breast cancer	Overexpression	Oncogene	Proliferation, invasion, cell growth	EZH2, CD44, ABCG2, miR-216-5p, Nanog, SOX2, Oct4, RXRA, GSK3β, PIK3CA, PI3K/AKT signaling pathway	31-33
	Downregulation	Suppressor	Metastasis, migration, EMT progression	CDK1-EZH2, TGF-β1, HDAC3, RUN2	29, 30
Lung cancer	Overexpression	Oncogene	Proliferation, invasion, migration, apoptosis, colony formation, cell cycle, cell growth, EMT progression	miR-216a, mTOR, miR-496, HMGA2, miR-758-3P, Sox4, miR-138, EZH2, p21	34-39
	Downregulation	Suppressor	Invasion, migration	TGF-β1	40
Glioma	Overexpression	Oncogene	Proliferation, invasion, metastasis, apoptosis, cell cycle,	miR-33a-5p, miR-645, RAB1A, β-catenin pathway, AXL, miR-33b-5p, miR-1-3p, miR-206, miR-613, NF-κB signaling pathway	41-44
Osteosarcoma	Overexpression	Oncogene	Proliferation, invasion, metastasis,	miR-33a-5p, AXL, PI3K-AKT signaling pathway, miR-335-5p, miR-1972, ROCK1, EZH2, p21, p27	45-48
cervical cancer	Overexpression	Oncogene	Proliferation, invasion, metastasis	miR-335-5p, miR-665, TGFBR1, ERK/SMAD pathway	49, 50
Bladder cancer	Overexpression	Oncogene	Proliferation, invasion, metastasis, malignant phenotype, EMT progression	miR-149, MSI2, IL-11-STAT3 signaling, cyclin D1, PLAU, LRPPRC	51, 52
Prostate cancer	Overexpression	Oncogene	Proliferation, invasion, metastasis, migration, malignant phenotype, EMT progression	TIM2/3, EZH2, androgen-AR signaling pathway, miR-34a-5p, JAG1	53, 54
Nasopharyngeal carcinoma	Overexpression	Oncogene	Proliferation, invasion, metastasis, migration, apoptosis, cell growth	HIF-1α, NF90/NF45, AKT, PTEN, IL-6, STAT3, JAK1, EZH2	55-58
Esophageal squamous cell carcinoma	Overexpression	Oncogene	Proliferation, invasion, migration, apoptosis	-	60
Ovarian cancer	Overexpression	Oncogene	Proliferation, invasion, migration	IGF2	61
Retinoblastoma	Overexpression	Oncogene	Proliferation, invasion, migration, EMT progression	N-cadherin, Vimentin, miR-34c, miR-613, MMP-9	62

## Abbreviations

LncRNAs: long noncoding RNAs
MALAT1: metastasis-associated lung adenocarcinoma transcript 1
NKILA: NF-KappaB interacting lncRNA
DANCR: differentiation antagonizing non-protein coding RNA
ANCR: anti-differentiation non-coding RNA
TNM: tumor-node-metastasis
EZH2: enhancer of zeste homolog 2
DDP: cisplatin
MDR: multidrug resistance
OS: overall survival
DFS: disease-free survival
HSP27: heat shock protein 27
PFS: progression-free survival
ceRNA: competing endogenous RNA
HMGA2: high mobility group AT-hook 2
EMT: epithelial-mesenchymal transition
TGFBR1: transforming growth factor beta receptor 1
MSI2: musashi RNA binding protein 2
LRPPRC: leucine-rich pentatricopeptide repeat containing
DTX: docetaxel
IGF2: insulin-like growth factor
PRC2: Polycomb Repressive Complex
H3K27me3: the trimethylation of Lys-27 of histone H3
CTNNB1: catenin beta 1
ILF3: interleukin enhancer binding factor 3
